# HIV-1 capsid exploitation of the host microtubule cytoskeleton during early infection

**DOI:** 10.1186/s12977-021-00563-3

**Published:** 2021-07-06

**Authors:** Mojgan H. Naghavi

**Affiliations:** Department of Microbiology-Immunology, Northwestern University Feinberg School of Medicine, Chicago, IL USA

**Keywords:** HIV-1, Capsid, Cytoskeleton, Microtubules, +TIPs, Motors, Trafficking, Uncoating

## Abstract

Microtubules (MTs) form a filamentous array that provide both structural support and a coordinated system for the movement and organization of macromolecular cargos within the cell. As such, they play a critical role in regulating a wide range of cellular processes, from cell shape and motility to cell polarization and division. The array is radial with filament minus-ends anchored at perinuclear MT-organizing centers and filament plus-ends continuously growing and shrinking to explore and adapt to the intracellular environment. In response to environmental cues, a small subset of these highly dynamic MTs can become stabilized, acquire post-translational modifications and act as specialized tracks for cargo trafficking. MT dynamics and stability are regulated by a subset of highly specialized MT plus-end tracking proteins, known as +TIPs. Central to this is the end-binding (EB) family of proteins which specifically recognize and track growing MT plus-ends to both regulate MT polymerization directly and to mediate the accumulation of a diverse array of other +TIPs at MT ends. Moreover, interaction of EB1 and +TIPs with actin-MT cross-linking factors coordinate changes in actin and MT dynamics at the cell periphery, as well as during the transition of cargos from one network to the other. The inherent structural polarity of MTs is sensed by specialized motor proteins. In general, dynein directs trafficking of cargos towards the minus-end while most kinesins direct movement toward the plus-end. As a pathogenic cargo, HIV-1 uses the actin cytoskeleton for short-range transport most frequently at the cell periphery during entry before transiting to MTs for long-range transport to reach the nucleus. While the fundamental importance of MT networks to HIV-1 replication has long been known, recent work has begun to reveal the underlying mechanistic details by which HIV-1 engages MTs after entry into the cell. This includes mimicry of EB1 by capsid (CA) and adaptor-mediated engagement of dynein and kinesin motors to elegantly coordinate early steps in infection that include MT stabilization, uncoating (conical CA disassembly) and virus transport toward the nucleus. This review discusses recent advances in our understanding of how MT regulators and their associated motors are exploited by incoming HIV-1 capsid during early stages of infection.

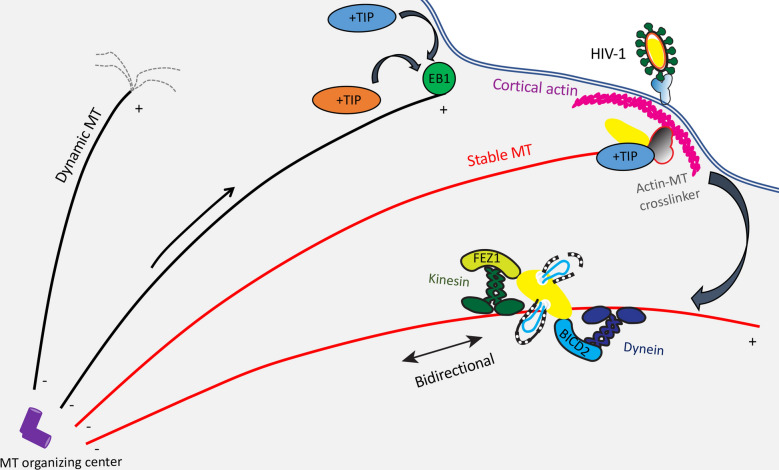

## Host cytoskeleton

The host cytoskeleton is a complex network of dynamic filaments radiating throughout the cell. The organization and function of cytoskeletal networks play important roles in cell shape, motility, polarity, or division and has been implicated in a wide range of diseases including neuronal disorders, muscle myopathies, various types of cancers, as well as viral infections [1–4]. These dynamic filaments are divided into three types: microfilaments, intermediate filaments and MTs. Microfilaments are the thinnest filaments in the cytoskeleton that are mostly composed of actin and are therefore also referred to as actin filaments. Intermediate filaments, which their name implies are in-between the size of microfilaments and MTs, help cells maintain their shape, bear tension and provide cellular structural support. Unlike intermediate filaments, microfilaments and MT cytoskeletons are polarized filaments responsible for active directional transport of a variety of cargos within the cell including viruses. Actin mediated transport is driven by the myosin family of motors, but can also occur via actin nucleation and/or polymerization regulated by Rho-GTPase signaling pathways [5]. Actin filaments mediate short distance motility of cargos most frequently at the cell periphery, while MTs are cytoskeletal highways responsible for long distance transport of cargos throughout the cell.

**Fig. 1:**
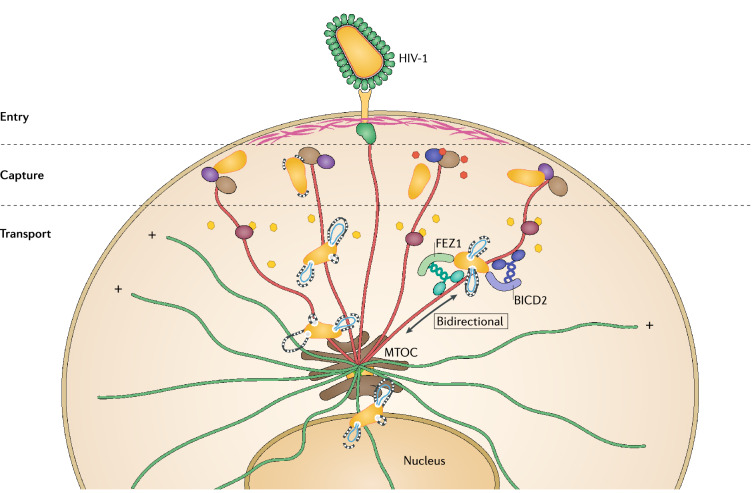
HIV-1 interactions with the host cell cytoskeleton during early infection. Entry. Upon entry by fusion with specific receptors at the plasma membrane, incoming HIV-1 cores penetrate through cortical actin (pink) and are deposited in the cytoplasm. Soon after entry, HIV-1 induces microtubule (MT) stabilization (shown as red filaments), which is regulated in part by actin-MT cross-linking proteins (green). Capture. Fusion of HIV-1 into the cytosol releases matrix (MA) protein (orange) that is captured by a complex of MT plus-end tracking proteins (+TIPs) consisting of the MT end-binding protein (EB1) (light brown) and Kif4 (dark blue) at the tip of MTs (+) to induce MT stabilization. The incoming conical capsid (yellow) is also captured with the EB1-associated actin-MT crosslinkers Dia1/2 (purple) to induce additional MT stabilization. The SxIP containing EB1-associated +TIPs CLIP170 and CLASP2 (dashed lines around the capsid) bind to and stabilize capsid, perhaps via recognition of local capsid lattice ruptures induced by the onset of reverse transcription, to upload the incoming cores from cortical actin onto stable MTs. Transport. The capsid binds MT motor adaptors BICD2 and FEZ1 to bridge viral particles to dynein (dark blue) and kinesin-1 (green) motors, respectively, to mediate their long-range bidirectional transport on stable MTs towards the nucleus. As the conical capsid moving along stable MTs loses small patches of CA to accommodate the outgrowing reverse transcribing viral genome (blue), CA protein release likely further increases MT stabilization over time via interactions with MT associated proteins MAP1A/S (dark red). Incoming cores accumulate at the perinuclear MT organizing center (MTOC) prior to nuclear entry by an as-yet unknown transport mechanism

MTs are composed of polarized heteropolymeric chains of α/β tubulin with their minus-ends anchored at a perinuclear MT organizing center (MTOC) and their plus-ends pointing toward the cell periphery, where they can interact with cortical actin (a highly dense layer of actin filaments underneath the plasma membrane) (Fig. 1) [6, 7]. The inherent structural polarity of the MTs is sensed by MT motors so that the single cytoplasmic dynein motor moves toward MT minus-ends (retrograde) and most kinesin motors move towards the plus-ends (anterograde). In most cell types, the majority of MTs are highly dynamic with their plus-ends undergoing rapid phases of growth and shrinkage, a behavior known as dynamic instability. The dynamic nature of MTs allows sensing of intracellular sites where their plus ends will interact with targets through a process often referred to as “search and capture”. While capture can initiate cargo transport in other cases, upon encountering targets such as regions of the cell cortex, organelles or cargos and in response to specific signals, the MT filament becomes locally stabilized. Given their longevity (t1/2 > 1 h as opposed to t1/2 of 5–10 min in the case of dynamic MTs), stable MTs acquire post-translational modifications including acetylation or detyrosination of α-tubulin. While tubulin acetylation that arises in the inner lumen of the MT filament was recently shown to confer mechanical strength, detyrosination on the outer surface does not impart stability but instead enables preferential binding of kinesin motors [8, 9]. This motor selectivity combined with their longevity allows stable MTs to act as ideal tracks for long-range transport of specific cargos during processes such as cell polarization [8–10]. MT behavior and stability is regulated by a range of MT-associated proteins (MAPs) that includes motors and MT regulators. While many nonmotor MAPs can promote MT stabilization by binding along the filament lattice, a highly specialized subset of MAPs known as end-binding (EB) proteins (comprising 3 family members EB1, EB2 or EB3) specifically recognize guanosine triphosphate-tubulin that is transiently present at the growing MT plus-ends, and thereby track growing MT tips [11]. The N-terminal region of EB proteins is sufficient to track MT plus-ends and promote MT polymerization, while their C-terminal domains mediate EB dimerization and interaction with other proteins including other MT plus-end tracking proteins (+TIPs). Although many +TIPs can directly bind MTs, the majority of +TIPs utilize CAP-Gly or SxIP motifs to bind to EB proteins in order to accumulate at MT plus-ends, making EBs the master regulators of MT plus-end behavior and function [11]. Recruitment of other +TIPs by EBs creates functional modules at the growing MT ends to mediate changes in MT dynamics, including signaling through Rac1, glycogen synthase kinase 3β (GSK3β) and RhoA-diaphanous (Rho-Dia) pathways. Some of these signaling pathways also trigger localized MT stabilization at specific subcellular sites, often to drive cell polarization during development, differentiation and cell migration. Interestingly, actin remodeling is also controlled by some of these signals, highlighting the importance of these pathways in coordinating overall cytoskeletal dynamics in a variety of contexts including virus infection.

## Interplay between HIV-1 capsid and host cytoskeletal regulatory factors

Given its size, it is perhaps not surprising that HIV-1, similar to many other viruses, has evolved means to exploit host cytoskeletal transport networks in order to facilitate its movement across the dense and viscous cytosolic environment which greatly impedes diffusion of large molecules. Earlier work showed that interaction of incoming HIV-1 with both actin and MT cytoskeleton alter the organization of these networks, while chemicals that disrupt these transport networks suppress viral infection. These reports suggested that for a successful infection, HIV-1 not only exploits the existing transport systems within infected cells but is also likely to actively manipulate them.

## Exploitation of actin cytoskeleton

HIV-1 has evolved various strategies to not only overcome the physical barrier imposed by the cortical actin to entry into the cell but to actively exploit the actin network and its associated factors to facilitate early infection. This includes surfing on the cell surface and entry of viral particles at active actin remodeling areas [12], mediating actin-rearrangements either directly using viral proteins [13, 14] (reviewed in [15]) or by exploiting host actin regulatory proteins through control of signaling pathways [16–21], or direct interaction with actin-binding proteins [22], or by counteracting inhibition of actin polymerization [23]. Moreover, podosomes (small dynamic actin-driven adhesion microdomains) were recently shown as a novel route for entry of HIV-1 into macrophages [24]. While HIV-1 induced actin remodeling is critical for short-range movement of the incoming viral cores at the cell periphery, this review focuses on the intracellular role of cytoskeletal networks that are targeted by HIV-1 capsid. For broader information readers are directed to more extensive reviews on this subject [15, 25].

## Exploitation of actin-MT cross-linkers

Once inside the cell, similar to many other viruses, HIV-1 exploits cortical actin for short-distance transport of incoming viral cores from the peripheral regions of the cell onto the MT network for long-ranged transport to the nuclear periphery [26, 27]. Earlier studies using actin depolymerizing agents suggested that actin-based motility was important either for cytoplasmic entry of incoming HIV-1 cores [17], for establishment of an infectious viral complex [28] and more recently for early infection and transport to the nucleus in T cells [29]. Interestingly, some of these studies suggest that MTs only play a very minor role in infection [29]. However, this conclusion was based on treating cells with MT modulators but removing them prior to infection. Given that many modulators, such as nocodazole, are rapidly reversible and that MTs rapidly re-polymerize within minutes of their removal, and that nocodazole does not affect stable MTs that are utilized by HIV-1 as discussed below, it remains uncertain whether MTs are truly not important in T cells. Indeed, depletion of +TIPs and interfering with MT motors has been shown to affect early infection of Jurkat T cells [30, 31], suggesting that MTs are indeed important in T-cells. In the Burkrinskaya et al. study, a direct interaction of HIV-1 matrix (MA) with actin (but not tubulin) was suggested to mediate localization of reverse transcription complex (RTC) on actin microfilaments resulting in promotion of the process of reverse transcription within the complex. The same study also showed that while MA within the RTC associated with actin cytoskeleton, CA (the major component of the incoming viral cores) did not exhibit any significant binding [28]. Later live cell microscopy imaging not only supported this proposed initial short-range actin-driven motility by showing MT-independent trafficking of incoming HIV-1 cores at the cell periphery, but also indicated a direct association of CA proteins with the MT cytoskeleton [32]. These discoveries suggested that incoming HIV-1 cores, similar to all other viruses that enter the cell by fusion into the cytosol at the plasma membrane, has to transit from the actin network to MTs, a hand-off process that often involves several host and viral proteins [27]. To this end, while relatively little is known about the proteins associated with incoming HIV-1 cores, a growing number of host factors that regulate actin-MT cross-linking and positively or negatively affect infection have been reported. Among these, focal adhesion proteins are required during early stages of HIV-1 infection [33], while the ezrin-radixin-moesin (ERM) family members, moesin and ezrin function as negative regulators of early post-entry infection, before or at the initiation of reverse transcription [34–36]. Given that these actin-MT cross-linkers also regulate MT stabilization, combined with the importance of stable MTs during early post-entry HIV-1 infection (discussed in more detail below), indicates that the effects of these factors on early infection likely reflect their ability to positively (focal adhesion proteins) or negatively (ERMs) regulate stable MT formation. Follow up studies further identified a novel moesin interacting factor PDZ domain-containing protein 8 (PDZD8) which not only induced MT stabilization and promoted early infection at the initiation of reverse transcription [37], but also bound to HIV-1 CA and affected the stability of incoming core [38] (Table 1). However, follow-up studies from the same lab showed that CRISPR-CAS9 knockout of PDZD8 did not significantly impact HIV-1 infection [39]. While the underlying reason for this apparent discrepancy remain unclear, it is possible that long-term knockout of PDZD8 requires the evolution of compensatory processes for cell survival and which negate the effect of PDZD8 loss on infection.

**Table 1:** MT regulatory factors that interact with HIV-1 capsid and their effects on early stages of infection

Gene name (NCBI)^a^	Gene ID (NCBI)^a^	Early steps of HIV-1 life cycle affected	References
PDZD8: PDZ domain containing protein 8	118987	Reverse transcription and capsid stability	[37, 38]
BICD2: Bicaudal D homology 2	23299	Retrograde trafficking and uncoating	[31, 97]
FEZ1: Fasciculation and elongation protein zeta 1	9638	Retrograde trafficking and uncoating	[69, 96]
MARK2: MT affinity regulating kinase 2	2011	Retrograde trafficking and uncoating	[69]
MAP1A: MT associated protein 1A	4130	Nuclear translocation	[69]
MAP1S: MT associated protein 1S	55201	Nuclear translocation	[64]
DIAPH1 (Dia1): Diaphanous related formin 1	1729	Retrograde trafficking, uncoating and reverse transcription	[42]
DIAPH2 (Dia2):Diaphanous related formin 2	1730	Retrograde trafficking, uncoating and reverse transcription	[42]
CLASP2: Cytoplasmic linker associated protein 2	23122	Retrograde trafficking	[30]
CLIP170: Cytoplasmic linker protein 170	6249	Retrograde trafficking and uncoating	[75]

^***a***^*NCBI* National Center for Biotechnology Information

In addition to PDZD8, other actin and actin-MT cross-linking proteins have more recently been implicated in regulation of uncoating and reverse transcription. Diaphanous-related formins (DRFs) Dia1 and Dia2, which control both actin nucleation and MT stabilization to coordinate cytoskeletal remodeling [40, 41], were recently found to not only facilitate HIV-1 induced MT stabilization and early transport but also to bind incoming viral cores and regulate uncoating and reverse transcription independently of their MT regulatory functions [42] (Table 1). Notably, the ability of DRFs to stabilize MTs and interact with incoming capsid is genetically separable, and the latter function, similar to PDZD8, correlates with the presence of coiled-coil domains in portions of DRFs that can regulate uncoating. As such, DRFs appear to couple HIV-1 capsid disassembly with the induction of MT stabilization and trafficking to the nucleus. Collectively, these findings uncover the role of actin-MT crosslinkers in the transition of incoming HIV-1 cores from peripheral actin to the MT network while also highlighting their specific roles in the poorly understood process of uncoating.

## The contentious topic of cytoplasmic versus nuclear uncoating

Several actin-MT crosslinkers discussed above, as well as MT regulators and motors discussed below, influence HIV-1 uncoating and reverse transcription. However, there are two very differing views in the HIV-1 field, one of which contends that uncoating does not occur in cytoplasm but instead occurs only after entry into the nucleus [43–45]. Intricately coupled, HIV-1 reverse transcription is known to promote conical CA disassembly or uncoating [46–49], while formation of viral dsDNA genome has been proposed to increase pressure that can rupture the viral capsid locally [50]. Supporting these findings, reconstitution of endogenous reverse transcription in a cell-free system followed by cryo-electron tomographic imaging has recently suggested that capsid uncoating does not occur in an all-or-none fashion but rather as a “local lattice rupture” where the initial stages involve the loss of relatively small surface patches, through which loops of the growing viral double-stranded cDNA could extrude [51]. Although these recent *in vitro* studies do not address the hotly debated question of when and where reverse transcription and uncoating occur in infected cells [52–55], they are in line with the thinking of several groups that suggest that reverse transcription and uncoating are complex processes that begin in the cytoplasm, termed “partial uncoating”, and complete inside the nucleus [32, 42, 46, 47, 56–75]. Indeed, partial loss of CA protein during cytoplasmic transport of HIV-1 cores to the nucleus has been visualized by the Hope lab for particles that go on to successfully deliver and express reporter genes [47]. Fully intact viral cores are also physically too large to pass through nuclear pores and recent structural studies suggest that some degree of loss of CA protein and/or structural changes are required for nuclear entry of viral cores. Remodeled viral CA complexes associated with the retrotranscribed DNA were observed at the nuclear membrane [76], while radial constriction of the capsid accommodated by local lattice ruptures were suggested as a model for how reverse transcribing viral cores maintain the majority of their CA subunits as they pass through nuclear pore complexes (NPC) [51]. However, very recent structural analyses suggest that NPCs in infected T cells are sufficiently wide for transport of intact HIV-1 capsid [45]. Whether this is a T cell-specific phenomenon and occurs for all or just some of the viral cores that ultimately infect the cell remains unclear. While cytoplasmic versus nuclear uncoating remains a hotly debated topic, it would seem most likely that HIV-1 cores are simply capable of undergoing either process and in some instances can enter the nucleus intact; the contentious camps are likely arguing two sides of the same coin. Regardless, as discussed below, recent work on MT regulators adds further support for the notion that partial uncoating in the cytoplasm is functionally important for early infection.

## Exploitation of MTs and MT regulators

Viruses often use different strategies to modulate MT dynamics to facilitate their transport to the nucleus [77–79]. HIV-1 does this by rapid induction of MT stabilization soon after the entry into the cell. Initial studies found that incoming HIV-1 cores localized on a specific subset of stable MTs, which were resistant to the MT depolymerizing agents such as nocodazole [80]. The MA protein of incoming HIV-1 particles was further shown to target a novel EB1-associated +TIP, Kif4 [81], to rapidly induce MT stabilization and facilitate the delivery of viral particles to the nucleus (Fig. 1) [80]. Indeed, this report not only provided the first demonstration of a function for +TIPs in infection by any virus, but also changed our view of the role of stable MTs in infection, demonstrating that earlier studies suggesting MTs were not important for HIV-1 infection based solely on the use of nocodazole, was in fact due to HIV-1’s use of stable nocodazole-resistant MT filaments.

Subsequent studies showed that HIV-1 exploits a number of EB1-associated +TIPs in a much broader fashion to stabilize MTs and control both the trafficking and uncoating of incoming viral cores (Fig. 1). The initial induction of stable MTs mediated by HIV-1 MA that is released into the cell is further enhanced by incoming capsid targeting a second EB1-associated +TIP complex consisting of the actin-MT cross-linking formins Dia1 and Dia2 [42]. This likely underlies a strategy for amplification of the levels of stable MTs as the virus proceeds through early infection. Intriguingly, as mentioned above, HIV-1 does not appear to utilize the known actin-regulatory function of these formins that operates in uninfected cells, but instead the capsid binds Dia1/2 to couple uncoating and stable MT-based viral movement. Other examples of EB1-associated +TIPs that bind HIV-1 capsids and regulate early infection include the cytoplasmic linker protein (CLIP)-associated protein 2 (CLASP2), a key regulator of cortical capture and MT stabilization [82, 83]. CLASP2 was found to bind incoming HIV-1 capsid through its N-terminal half while, independently, a critical domain in its C-terminus facilitated trafficking of incoming cores via the induction of MT stabilization [30] (Table 1). As such, HIV-1 exploits distinct functions of several +TIPs to coordinate early stages of infection through both MA protein and viral cores themselves.

Beyond +TIPs, interaction of HIV-1 CA with MT-associated proteins MAP1A and MAP1S has also been proposed to promote retrograde viral trafficking by facilitating MT stabilization and/or tethering incoming viral capsids to MT filaments [64] (Table 1). Given that these MAPs do have not have motor activity, their ability to mediate MT stabilization most likely provides more long-lived and potentially selective networks for cytoplasmic transport of HIV-1. While capsid binders such as MAPs and EB1-associated +TIPs increase the formation of stable MTs following infection, other factors such as suppressor of G2 allele of *skp1* (SUGT1) that don’t directly associate with incoming viral cores also maintain the stability of MT plus-ends to promote retrograde trafficking and replication of HIV-1 in lymphocytes and macrophages [84]. Other examples include how the mechanistic target of rapamycin (mTOR) mediates MT stabilization to facilitate early HIV-1 infection in T cells [85]. Together, these findings highlight the extent to which incoming HIV-1 proteins, in particular capsids target MT regulatory factors to ensure MT stabilization required during early stages of infection.

## HIV-1 capsid mimicry of EB1

Recent work has shed light on how HIV-1 particles are capable of engaging several different classes of +TIPs to regulate MT dynamics together with trafficking and uncoating of viral cores, which occurs at least in part through mimicry of EB1. Beyond its direct role in regulating MT dynamics that control infection, EB1 was also found to indirectly contribute to early HIV-1 infection by delivering +TIPs such as cytoplasmic linker protein-170 (CLIP170) to the cell periphery [75] (Table 1). CLIP170 was shown to function differently to EB1 or other previously identified +TIPs discussed above, in that it regulates infection without significantly affecting the formation of stable MTs. Given its established role in actin-MT linkage and cargo capture [86, 87], CLIP170 may be important for uploading of incoming HIV-1 cores from actin onto MTs to initiate transport, similar to how herpes simplex virus type 1 (HSV-1) do not randomly engage MTs but rather are captured by specific MT plus-ends-associated complexes to initiate retrograde transport [88]. In the case of HIV-1, CLIP170 also influences uncoating despite not affecting MT stability [75], which again highlights the diverse roles played by +TIPs at the ends of MT filaments in mediating various early steps in the infectious lifecycle. Notably, this study showed that EB1 functions indirectly by regulating MT dynamics and delivering +TIPs that bind HIV-1 particles to the periphery, but does not itself directly bind HIV-1 particles. By contrast, CLIP170 bound and colocalized with incoming cores, while its peripheral localization was required to promote early HIV-1 infection. CLIP170 bound HIV-1 capsid in a pattern that is distinct [75] from currently known capsid-binding cofactors such a cyclophilin A (CypA) [89]. Unlike CypA, CLIP170 also binds *in vitro* assembled cores derived from the CA mutant, R18L [90], which suggests that CLIP170 binding may be influenced by capsid curvature or the local composition of hexamers and pentamers, or openings in *in vitro* assembled cores that mimic a partially uncoated state.

In understanding why a growing number of +TIPs, but not EB1 itself, bound to incoming HIV-1 cores, these studies further revealed the existence of an EB-like +TIP-binding motif within the conserved major homology domain (MHR) of HIV-1 CA that binds SxIP motifs found in several +TIPs [75]. Given that the MHR is localized internally it is likely exposed by partial uncoating at early stages after entry, and this exposure may be further influenced by pentamer configurates with hexamers based on specific binding to R18L mutants discussed above. Given the known instability of HIV-1 capsid with higher levels of pentamers [91], it is tempting to envisage a scenario where pentamer-rich regions might initiate the first stages of capsid rupture/disassembly and are recognized by +TIPs to actually prevent complete uncoating by stabilizing partially uncoated cores as they travel towards the nucleus (Fig. 1). This would agree with reports using live cell imaging approaches that show partial uncoating of infectious viral complex soon after entry into the cytoplasm [47], yet capsid remains largely intact all the way into the nucleus [43–45, 72]. This model also supports recently proposed local capsid fracturing model during the initial stages of uncoating both in a cell free system [51] as well as for the capsid inhibitor PF74 in infected cells, where rapid uncoating is observed in the absence of capsid binders, suggesting that partially uncoated states with the capsid largely intact do not exist unless stabilized by cellular factors in infected cells [71]. Given that MT regulatory proteins such as DRFs [42] also enhance reverse transcription, it is possible that their binding to incoming cores might also be how the growing viral double-stranded cDNA that is extruded through lattice openings is shielded from cytoplasmic sensors [92, 93].

Intriguingly, the divergent MHR sequence [94] of simian immunodeficiency virus (SIV) has only weak EB1-mimetic activity, while murine leukemia virus (MuLV) lack this activity, which correlates with the ability of these retroviruses to induce MT stabilization and engage CLIP170 to facilitate early infection, suggesting divergent capsid-based EB1 mimicry across retroviral species [75]. Collectively, these findings highlight how +TIP binding motif mimicry within HIV-1 capsid creates functional modules for various +TIPs found at the ends of growing MTs, enabling the virus to not only induce global MT stabilization but also coordinate different steps of early infection.

## Exploitation of MT motors and their associated adaptors

Like a number of other viruses [77–79], earlier studies of HIV-1 movement in living cells demonstrated that incoming HIV-1 particles exhibit bi-directional MT-based motility that suggested their association with both dynein (inward-directed) and kinesin (outward-directed) MT motors as they travel towards the nucleus (Fig. 1) [32, 95]. However, unlike many other viruses that bind MT motors directly, HIV-1 particles had not been found to bind to either dynein or kinesin motors. Despite this, in recent years, a number of significant advances have been made in our understanding of how incoming HIV-1 cores engage MT motors. Incoming HIV-1 capsids were shown to associate with the kinesin-1 adaptor fasciculation and elongation factor zeta-1 (FEZ1) to enable the virus to regulate its retrograde (towards the nucleus) motility [96] (Table 1). Although kinesin-1 is normally an outward motor, capsid-bound FEZ1 regulates the balance of motor activity to ensure net forward movement to the nucleus for efficient infection [96]. Soon after this, Bicaudal D homology 2 (BICD2) was identified as a capsid-specific adaptor for dynein-mediated retrograde transport of incoming viral cores [31, 97] (Table 1). BICD2 is a dynein cargo adaptor and activator, which also bridges with dynein-activating complex, dynactin [98]. To this end, studies show that other components of dynein-dynactin complex including dynein light chain proteins, dynein heavy chain and specific core dynactin subunits also facilitate different stages of early HIV-1 infection [31, 65, 97, 99, 100]. However, unlike many other viruses [77] and several cellular cargos [101], HIV-1 does not require the core dynein adaptor subunit dynactin-1 (DCTN1 or p150Glued) for early infection [31]. The underlying reason for why HIV-1 favors BICD2-based engagement of dynein instead of DCTN1 remains unknown. Combined, these findings suggested that instead of directly interacting with motors, HIV-1 uses unusual motor adaptor proteins to both engage and regulate motor activities needed for transport of incoming viral cores. Given the importance of HIV-1 induced MT stabilization during early infection, along with the fact that post-translationally modified stable MT filaments have a preference for kinesin-1 motors discussed above, it seems likely that HIV-1 has evolved this FEZ1 adaptor-based mechanism to control kinesin-1 activity and facilitate bidirectional movements on stable MTs in order to reach the nucleus. In line with the fact that HIV-1 reverse transcription and uncoating are intricately intertwined with bidirectional transport on MTs, inhibition of either MT motor or motor adaptors also affected HIV-1 uncoating, suggesting that disassembly of capsid may be aided by the tug-of-war forces generated by opposing motors [62, 96, 97].

Subsequent studies uncovered the underlying mechanisms by which HIV-1 locally controls FEZ1 activity to promote early infection. It became evident that phosphorylation of FEZ1 at Serine 58 (S58), which regulates FEZ1’s interaction with kinesin-1 heavy chain (Kif5B) [102], was required for the nuclear translocation and uncoating of HIV-1 capsids [69] (Table 1). Moreover, the microtubule associated regulatory kinase 2 (MARK2) was identified as the host kinase [103] that locally regulated FEZ1 activity on HIV-1 cores, and that this played a critical role in controlling both bidirectional motility and uncoating of incoming viral cores towards the nucleus [69]. Structural analysis further identified FEZ1 as one of the highest-affinity HIV-1 capsid-interacting proteins identified to date and revealed how FEZ1 specifically bound to the central capsid hexamer pore to promote trafficking of incoming HIV-1 cores to the nucleus in infected cells [104, 105]. These findings not only uncovered the structural basis for FEZ1’s interactions with HIV-1 capsids but also demonstrated how, by recruiting both the motor adaptor FEZ1 and its regulatory kinase MARK2 to viral cores, HIV-1 carefully controls kinesin-1 activity.

MT motors transport the incoming viral cores only as far as the perinuclear MTOC. Like many other viruses [77–79], HIV-1 has been observed to accumulate at the centrosome, the most dominant MTOC in many cell types, prior to nuclear entry (Fig. 1) [32]. Moreover, HIV-1 centrososmal accumulation has been suggested to be via direct interaction of integrase (IN) with MAPs such as the dynein light chain protein DYN2P and the centrosomal protein Stu2p [106, 107]. Intriguingly, HIV-1 has been reported to persist in quiescent T cells as a stable centrosome-associated pre-integration intermediate for several weeks, and it can return to productive infection upon T cell activation [108]. How HIV-1 makes the final transition from the perinuclear centrosome to the nuclear membrane is among the least understood processes during early trafficking. This is a brief anterograde movement from the MT nucleation site to the nucleus and the requirements for active transport across this region remains poorly understood. Earlier studies suggested that HIV-1 switches from fast MT-dependent to slower and possibly actin-dependent motions nearing the end of its journey to the nucleus [95]. However, more recently Kinesins have been implicated in docking and uncoating of viral cores at the nucleus [109], while structural analyses indicate a significant number of intact HIV-1 capsids in close proximity to MTs and NPCs [45]. Despite this, the mechanism by which incoming capsids are transported from the perinuclear centrosome to the nucleus remains underexplored.

## Conclusions

Although HIV-1 has long been known to utilize the host cell cytoskeleton throughout its lifecycle, a significant number of recent advances cited here illustrate how HIV-1 capsid functions to directly control and exploit MT behavior and function to facilitate early infection. We now understand that HIV-1 capsid binds motor adaptors, including direct engagement of FEZ1 to regulate kinesin-1 activity for retrograde transport. Moreover, HV-1 capsids bind a surprisingly wide range of specialized regulatory proteins to induce MT stabilization, generating MT subsets that have increased longevity and higher affinity for MT motors for efficient trafficking towards the nucleus. Moreover, in line with the nature of intricately intertwined processes of early infection, it is becoming increasingly apparent that incoming capsids take advantage of various functions of these specialized MT regulators to coordinate timing of their disassembly with the onset of MT stabilization and MT-based bidirectional transport towards the nucleus. Combined with structural studies revealing remodeling of capsid proteins around the viral DNA required for nuclear entry and live cell imaging approaches that show partial uncoating of infectious virus in the cytoplasm, the role of MT regulatory proteins in regulating uncoating and in particular, proteins such as CLIP170 that recognize internal MHR domains, lends further weight to the concept of partial uncoating in the cytoplasm. Indeed, in line with this model, recent *in vitro* capsid structural studies elegantly demonstrate a continuum of reverse transcribing capsid intermediates proposing loss of small surface patches to accommodate the growing vial DNA during the initial stages of capsid uncoating. Some suggest that the viral core must be intact in order to shield the viral genome from cytoplasmic sensors, yet the host factors that are thought to stabilize partially uncoated cores could equally shield viral DNA from the sensing machinery. As such, the continued study of how MAPs and motors regulate infection will undoubtedly provide valuable insights into these hotly debated topics in the future.

Understanding the molecular details by which capsid exploits specialized host MT networks, regulators and motors will not only provide important new insights into mechanisms of intracellular movement during HIV-1 infection, but will also have broader implications for our understating of how cells regulate MT dynamics and cargo trafficking. Furthermore, while tubulin-binding drugs currently used in chemotherapy are the “atom bomb” approach to targeting MTs and are quite toxic, more refined version of such drugs targeting highly specialized MT regulators could potentially be an attractive approach for development of new targeted therapeutic strategies to treat HIV-1. Indeed, a cell-permeable small peptide targeting EB3 which is structurally similar to EB1 was recently shown to suppress human cytomegalovirus (HCMV) infection [110]. Targeting host proteins with such specialized functions would also have the added benefit of avoiding the emergence of drug resistance commonly associated with targeting viral proteins.

## Data Availability

Not applicable.

## References

[1] Greber UF, Way M (2006). A superhighway to virus infection. Cell.

[2] Desiniotis A, Kyprianou N (2011). Significance of talin in cancer progression and metastasis. Int Rev Cell Mol Biol.

[3] Kuijpers M, Hoogenraad CC (2011). Centrosomes, microtubules and neuronal development. Mol Cell Neurosci.

[4] Tischfield MA, Cederquist GY, Gupta ML, Engle EC (2011). Phenotypic spectrum of the tubulin-related disorders and functional implications of disease-causing mutations. Curr Opin Genet Dev.

[5] Rottner K, Faix J, Bogdan S, Linder S, Kerkhoff E (2017). Actin assembly mechanisms at a glance. J Cell Sci..

[6] Kaverina I, Straube A (2011). Regulation of cell migration by dynamic microtubules. Semin Cell Dev Biol.

[7] Li R, Gundersen GG (2008). Beyond polymer polarity: how the cytoskeleton builds a polarized cell. Nat Rev Mol Cell Biol.

[8] Portran D, Schaedel L, Xu Z, Thery M, Nachury MV (2017). Tubulin acetylation protects long-lived microtubules against mechanical ageing. Nat Cell Biol.

[9] Xu Z, Schaedel L, Portran D, Aguilar A, Gaillard J, Marinkovich MP (2017). Microtubules acquire resistance from mechanical breakage through intralumenal acetylation. Science.

[10] Gundersen GG (2002). Evolutionary conservation of microtubule-capture mechanisms. Nat Rev Mol Cell Biol.

[11] Akhmanova A, Steinmetz MO (2015). Control of microtubule organization and dynamics: two ends in the limelight. Nat Rev Mol Cell Biol.

[12] Lehmann MJ, Sherer NM, Marks CB, Pypaert M, Mothes W (2005). Actin- and myosin-driven movement of viruses along filopodia precedes their entry into cells. J Cell Biol.

[13] Chazal N, Singer G, Aiken C, Hammarskjold ML, Rekosh D (2001). Human immunodeficiency virus type 1 particles pseudotyped with envelope proteins that fuse at low pH no longer require Nef for optimal infectivity. J Virol.

[14] Campbell EM, Nunez R, Hope TJ (2004). Disruption of the actin cytoskeleton can complement the ability of Nef to enhance human immunodeficiency virus type 1 infectivity. J Virol..

[15] Ospina Stella A, Turville S (2018). All-Round Manipulation of the Actin Cytoskeleton by HIV. Viruses.

[16] Del Real G, Jimenez-Baranda S, Lacalle RA, Mira E, Lucas P, Gomez-Mouton C (2002). Blocking of HIV-1 infection by targeting CD4 to nonraft membrane domains. J Exp Med.

[17] Iyengar S, Hildreth JE, Schwartz DH (1998). Actin-dependent receptor colocalization required for human immunodeficiency virus entry into host cells. J Virol.

[18] Jimenez-Baranda S, Gomez-Mouton C, Rojas A, Martinez-Prats L, Mira E, Ana Lacalle R (2007). Filamin-A regulates actin-dependent clustering of HIV receptors. Nat Cell Biol.

[19] Yoder A, Yu D, Dong L, Iyer SR, Xu X, Kelly J (2008). HIV envelope-CXCR4 signaling activates cofilin to overcome cortical actin restriction in resting CD4 T cells. Cell.

[20] Barrero-Villar M, Cabrero JR, Gordon-Alonso M, Barroso-Gonzalez J, Alvarez-Losada S, Munoz-Fernandez MA (2009). Moesin is required for HIV-1-induced CD4-CXCR4 interaction, F-actin redistribution, membrane fusion and viral infection in lymphocytes. J Cell Sci.

[21] Harmon B, Campbell N, Ratner L (2010). Role of Abl kinase and the Wave2 signaling complex in HIV-1 entry at a post-hemifusion step. PLoS Pathog.

[22] Yin W, Li W, Li Q, Liu Y, Liu J, Ren M (2020). Real-time imaging of individual virion-triggered cortical actin dynamics for human immunodeficiency virus entry into resting CD4 T cells. Nanoscale.

[23] Paoletti A, Allouch A, Caillet M, Saidi H, Subra F, Nardacci R (2019). HIV-1 envelope overcomes NLRP3-mediated inhibition of F-actin polymerization for viral entry. Cell Rep.

[24] Li W, Liu J, Liu Y, Li Q, Yin W, Wanderi KK (2021). HIV-1 uses dynamic podosomes for entry into macrophages. J Virol.

[25] Spear M, Guo J, Wu Y (2012). The trinity of the cortical actin in the initiation of HIV-1 infection. Retrovirology.

[26] Taylor MP, Koyuncu OO, Enquist LW (2011). Subversion of the actin cytoskeleton during viral infection. Nat Rev Microbiol.

[27] Walsh D, Naghavi MH (2019). Exploitation of cytoskeletal networks during early viral infection. Trends Microbiol.

[28] Bukrinskaya A, Brichacek B, Mann A, Stevenson M (1998). Establishment of a functional human immunodeficiency virus type 1 (HIV-1) reverse transcription complex involves the cytoskeleton. J Exp Med.

[29] Yoder A, Guo J, Yu D, Cui Z, Zhang XE, Wu Y (2011). Effects of microtubule modulators on HIV-1 infection of transformed and resting CD4 T cells. J Virol.

[30] Mitra S, Shanmugapriya S, Santos da Silva E, Naghavi MH (2020). HIV-1 exploits CLASP2 to induce microtubule stabilization and facilitate virus trafficking to the nucleus. J Virol.

[31] Carnes SK, Zhou J, Aiken C (2018). HIV-1 engages a dynein-dynactin-BICD2 complex for infection and transport to the nucleus. J Virol.

[32] McDonald D, Vodicka MA, Lucero G, Svitkina TM, Borisy GG, Emerman M (2002). Visualization of the intracellular behavior of HIV in living cells. J Cell Biol.

[33] Brown C, Morham SG, Walsh D, Naghavi MH (2011). Focal adhesion proteins talin-1 and vinculin negatively affect paxillin phosphorylation and limit retroviral infection. J Mol Biol.

[34] Naghavi MH, Valente S, Hatziioannou T, de Los Santos K, Wen Y, Mott C (2007). Moesin regulates stable microtubule formation and limits retroviral infection in cultured cells. EMBO J.

[35] Haedicke J, de Los Santos K, Goff SP, Naghavi MH (2008). The Ezrin-radixin-moesin family member ezrin regulates stable microtubule formation and retroviral infection. J Virol.

[36] Kubo Y, Yoshii H, Kamiyama H, Tominaga C, Tanaka Y, Sato H (2008). Ezrin, Radixin, and Moesin (ERM) proteins function as pleiotropic regulators of human immunodeficiency virus type 1 infection. Virology.

[37] Henning MS, Morham SG, Goff SP, Naghavi MH (2010). PDZD8 is a novel Gag-interacting factor that promotes retroviral infection. J Virol.

[38] Guth CA, Sodroski J (2014). Contribution of PDZD8 to stabilization of the human immunodeficiency virus type 1 capsid. J Virol.

[39] Zhang S, Sodroski J (2015). Efficient human immunodeficiency virus (HIV-1) infection of cells lacking PDZD8. Virology.

[40] Kuhn S, Geyer M (2014). Formins as effector proteins of Rho GTPases. Small GTPases.

[41] Ridley AJ (2015). Rho GTPase signalling in cell migration. Curr Opin Cell Biol.

[42] Delaney MK, Malikov V, Chai Q, Zhao G, Naghavi MH (2017). Distinct functions of diaphanous-related formins regulate HIV-1 uncoating and transport. Proc Natl Acad Sci USA.

[43] Burdick RC, Li C, Munshi M, Rawson JMO, Nagashima K, Hu WS (2020). HIV-1 uncoats in the nucleus near sites of integration. Proc Natl Acad Sci USA.

[44] Selyutina A, Persaud M, Lee K, KewalRamani V, Diaz-Griffero F (2020). Nuclear import of the HIV-1 core precedes reverse transcription and uncoating. Cell Rep.

[45] Zila V, Margiotta E, Turonova B, Muller TG, Zimmerli CE, Mattei S (2021). Cone-shaped HIV-1 capsids are transported through intact nuclear pores. Cell.

[46] Hulme AE, Perez O, Hope TJ (2011). Complementary assays reveal a relationship between HIV-1 uncoating and reverse transcription. Proc Natl Acad Sci USA.

[47] Mamede JI, Cianci GC, Anderson MR, Hope TJ (2017). Early cytoplasmic uncoating is associated with infectivity of HIV-1. Proc Natl Acad Sci USA.

[48] Yang Y, Fricke T, Diaz-Griffero F (2013). Inhibition of reverse transcriptase activity increases stability of the HIV-1 core. J Virol.

[49] Cosnefroy O, Murray PJ, Bishop KN (2016). HIV-1 capsid uncoating initiates after the first strand transfer of reverse transcription. Retrovirology.

[50] Rankovic S, Varadarajan J, Ramalho R, Aiken C, Rousso I (2017). Reverse transcription mechanically initiates HIV-1 capsid disassembly. J Virol.

[51] Christensen DE, Ganser-Pornillos BK, Johnson JS, Pornillos O, Sundquist WI (2020). Reconstitution and visualization of HIV-1 capsid-dependent replication and integration in vitro. Science.

[52] Ambrose Z, Aiken C (2014). HIV-1 uncoating: connection to nuclear entry and regulation by host proteins. Virology.

[53] Campbell EM, Hope TJ (2015). HIV-1 capsid: the multifaceted key player in HIV-1 infection. Nat Rev Microbiol.

[54] Yamashita M, Engelman AN (2017). Capsid-dependent host factors in HIV-1 infection. Trends Microbiol.

[55] Novikova M, Zhang Y, Freed EO, Peng K (2019). Multiple roles of HIV-1 capsid during the virus replication cycle. Virol Sin.

[56] Miller MD, Farnet CM, Bushman FD (1997). Human immunodeficiency virus type 1 preintegration complexes: studies of organization and composition. J Virol.

[57] Fassati A, Goff SP (2001). Characterization of intracellular reverse transcription complexes of human immunodeficiency virus type 1. J Virol.

[58] Arhel NJ, Souquere-Besse S, Munier S, Souque P, Guadagnini S, Rutherford S (2007). HIV-1 DNA Flap formation promotes uncoating of the pre-integration complex at the nuclear pore. EMBO J.

[59] Xu H, Franks T, Gibson G, Huber K, Rahm N, Strambio De Castillia C (2013). Evidence for biphasic uncoating during HIV-1 infection from a novel imaging assay. Retrovirology.

[60] Peng K, Muranyi W, Glass B, Laketa V, Yant SR, Tsai L (2014). Quantitative microscopy of functional HIV post-entry complexes reveals association of replication with the viral capsid. Elife.

[61] Zhou L, Sokolskaja E, Jolly C, James W, Cowley SA, Fassati A (2011). Transportin 3 promotes a nuclear maturation step required for efficient HIV-1 integration. PLoS Pathog.

[62] Lukic Z, Dharan A, Fricke T, Diaz-Griffero F, Campbell EM (2014). HIV-1 uncoating is facilitated by dynein and kinesin 1. J Virol.

[63] Hulme AE, Kelley Z, Okocha EA, Hope TJ (2015). Identification of capsid mutations that alter the rate of HIV-1 uncoating in infected cells. J Virol.

[64] Fernandez J, Portilho DM, Danckaert A, Munier S, Becker A, Roux P (2015). Microtubule-associated proteins 1 (MAP1) promote human immunodeficiency virus type I (HIV-1) intracytoplasmic routing to the nucleus. J Biol Chem.

[65] Jayappa KD, Ao Z, Wang X, Mouland AJ, Shekhar S, Yang X (2015). Human immunodeficiency virus type 1 employs the cellular dynein light chain 1 protein for reverse transcription through interaction with its integrase protein. J Virol.

[66] Chen NY, Zhou L, Gane PJ, Opp S, Ball NJ, Nicastro G (2016). HIV-1 capsid is involved in post-nuclear entry steps. Retrovirology.

[67] Francis AC, Marin M, Shi J, Aiken C, Melikyan GB (2016). Time-resolved imaging of single HIV-1 uncoating in vitro and in living cells. PLoS Pathog.

[68] Stultz RD, Cenker JJ, McDonald D (2017). Imaging HIV-1 genomic DNA from entry through productive infection. J Virol.

[69] Malikov V, Naghavi MH (2017). Localized phosphorylation of a kinesin-1 adaptor by a capsid-associated kinase regulates HIV-1 motility and uncoating. Cell Rep.

[70] Francis AC, Melikyan GB (2018). Single HIV-1 imaging reveals progression of infection through CA-dependent steps of docking at the nuclear pore, uncoating, and nuclear transport. Cell Host Microbe.

[71] Marquez CL, Lau D, Walsh J, Shah V, McGuinness C, Wong A (2018). Kinetics of HIV-1 capsid uncoating revealed by single-molecule analysis. Elife.

[72] Dharan A, Bachmann N, Talley S, Zwikelmaier V, Campbell EM (2020). Nuclear pore blockade reveals that HIV-1 completes reverse transcription and uncoating in the nucleus. Nat Microbiol.

[73] Ingram Z, Taylor M, Okland G, Martin R, Hulme AE (2020). Characterization of HIV-1 uncoating in human microglial cell lines. Virol J.

[74] Eschbach JE, Elliott JL, Li W, Zadrozny KK, Davis K, Mohammed SJ (2020). Capsid lattice destabilization leads to premature loss of the viral genome and integrase enzyme during HIV-1 infection. J Virol.

[75] da Santos Silva E, Shanmugapriya S, Malikov V, Gu F, Delaney MK, Naghavi MH (2020). HIV-1 capsids mimic a microtubule regulator to coordinate early stages of infection. EMBO J.

[76] Blanco-Rodriguez G, Gazi A, Monel B, Frabetti S, Scoca V, Mueller F (2020). Remodeling of the core leads HIV-1 pre-integration complex in the nucleus of human lymphocytes. J Virol.

[77] Radtke K, Dohner K, Sodeik B (2006). Viral interactions with the cytoskeleton: a hitchhiker's guide to the cell. Cell Microbiol.

[78] Dodding MP, Way M (2011). Coupling viruses to dynein and kinesin-1. EMBO J.

[79] Naghavi MH, Walsh D (2017). Microtubule Regulation and Function during Virus Infection. J Virol.

[80] Sabo Y, Walsh D, Barry DS, Tinaztepe S, de Los Santos K, Goff SP (2013). HIV-1 induces the formation of stable microtubules to enhance early infection. Cell Host Microbe.

[81] Morris EJ, Nader GP, Ramalingam N, Bartolini F, Gundersen GG (2014). Kif4 interacts with EB1 and stabilizes microtubules downstream of Rho-mDia in migrating fibroblasts. PLoS One.

[82] Akhmanova A, Hoogenraad CC, Drabek K, Stepanova T, Dortland B, Verkerk T (2001). Clasps are CLIP-115 and -170 associating proteins involved in the regional regulation of microtubule dynamics in motile fibroblasts. Cell.

[83] Efimov A, Kharitonov A, Efimova N, Loncarek J, Miller PM, Andreyeva N (2007). Asymmetric CLASP-dependent nucleation of noncentrosomal microtubules at the trans-Golgi network. Dev Cell.

[84] Allouch A, Di Primio C, Paoletti A, Le-Bury G, Subra F, Quercioli V (2020). SUGT1 controls susceptibility to HIV-1 infection by stabilizing microtubule plus-ends. Cell Death Differ.

[85] Taylor HE, Calantone N, Lichon D, Hudson H, Clerc I, Campbell EM (2020). mTOR Overcomes Multiple Metabolic Restrictions to Enable HIV-1 Reverse Transcription and Intracellular Transport. Cell Rep.

[86] Honnappa S, Okhrimenko O, Jaussi R, Jawhari H, Jelesarov I, Winkler FK (2006). Key interaction modes of dynamic +TIP networks. Mol Cell.

[87] Weisbrich A, Honnappa S, Jaussi R, Okhrimenko O, Frey D, Jelesarov I (2007). Structure-function relationship of CAP-Gly domains. Nat Struct Mol Biol.

[88] Jovasevic V, Naghavi MH, Walsh D (2015). Microtubule plus end-associated CLIP-170 initiates HSV-1 retrograde transport in primary human cells. J Cell Biol.

[89] Luban J, Bossolt KL, Franke EK, Kalpana GV, Goff SP (1993). Human immunodeficiency virus type 1 Gag protein binds to cyclophilins A and B. Cell.

[90] Ganser-Pornillos BK, von Schwedler UK, Stray KM, Aiken C, Sundquist WI (2004). Assembly properties of the human immunodeficiency virus type 1 CA protein. J Virol.

[91] Pornillos O, Ganser-Pornillos BK, Yeager M (2011). Atomic-level modelling of the HIV capsid. Nature.

[92] Rasaiyaah J, Tan CP, Fletcher AJ, Price AJ, Blondeau C, Hilditch L (2013). HIV-1 evades innate immune recognition through specific cofactor recruitment. Nature.

[93] Le Sage V, Mouland AJ, Valiente-Echeverria F (2014). Roles of HIV-1 capsid in viral replication and immune evasion. Virus Res.

[94] Tanaka M, Robinson BA, Chutiraka K, Geary CD, Reed JC, Lingappa JR (2016). Mutations of conserved residues in the major homology region arrest assembling HIV-1 Gag as a membrane-targeted intermediate containing genomic RNA and cellular proteins. J Virol.

[95] Arhel N, Genovesio A, Kim KA, Miko S, Perret E, Olivo-Marin JC (2006). Quantitative four-dimensional tracking of cytoplasmic and nuclear HIV-1 complexes. Nat Methods.

[96] Malikov V, Da Silva ES, Jovasevic V, Bennett G, Vieira DA, Schulte B (2015). HIV-1 capsids bind and exploit the kinesin-1 adaptor FEZ1 for inward movement to the nucleus. Nat Commun.

[97] Dharan A, Opp S, Abdel-Rahim O, Keceli SK, Imam S, Diaz-Griffero F (2017). Bicaudal D2 facilitates the cytoplasmic trafficking and nuclear import of HIV-1 genomes during infection. Proc Natl Acad Sci USA.

[98] Hoogenraad CC, Akhmanova A (2016). Bicaudal D family of motor adaptors: linking dynein motility to cargo binding. Trends Cell Biol.

[99] Pawlica P, Berthoux L (2014). Cytoplasmic dynein promotes HIV-1 uncoating. Viruses.

[100] Caly L, Kassouf VT, Moseley GW, Diefenbach RJ, Cunningham AL, Jans DA (2016). Fast track, dynein-dependent nuclear targeting of human immunodeficiency virus Vpr protein; impaired trafficking in a clinical isolate. Biochem Biophys Res Commun.

[101] Reck-Peterson SL, Redwine WB, Vale RD, Carter AP (2018). The cytoplasmic dynein transport machinery and its many cargoes. Nat Rev Mol Cell Biol.

[102] Chua JJ, Butkevich E, Worseck JM, Kittelmann M, Gronborg M, Behrmann E (2012). Phosphorylation-regulated axonal dependent transport of syntaxin 1 is mediated by a Kinesin-1 adapter. Proc Natl Acad Sci USA.

[103] Butkevich E, Hartig W, Nikolov M, Erck C, Grosche J, Urlaub H (2016). Phosphorylation of FEZ1 by microtubule affinity regulating kinases regulates its function in presynaptic protein trafficking. Sci Rep.

[104] Huang PT, Summers BJ, Xu C, Perilla JR, Malikov V, Naghavi MH (2019). FEZ1 is recruited to a conserved cofactor site on capsid to promote HIV-1 trafficking. Cell Rep.

[105] Summers BJ, Digianantonio KM, Smaga SS, Huang PT, Zhou K, Gerber EE (2019). Modular HIV-1 capsid assemblies reveal diverse host-capsid recognition mechanisms. Cell Host Microbe.

[106] Desfarges S, Salin B, Calmels C, Andreola ML, Parissi V, Fournier M (2009). HIV-1 integrase trafficking in S. cerevisiae: a useful model to dissect the microtubule network involvement of viral protein nuclear import. Yeast.

[107] de Soultrait VR, Caumont A, Durrens P, Calmels C, Parissi V, Recordon P (2002). HIV-1 integrase interacts with yeast microtubule-associated proteins. Biochim Biophys Acta.

[108] Zamborlini A, Lehmann-Che J, Clave E, Giron ML, Tobaly-Tapiero J, Roingeard P (2007). Centrosomal pre-integration latency of HIV-1 in quiescent cells. Retrovirology.

[109] Dharan A, Talley S, Tripathi A, Mamede JI, Majetschak M, Hope TJ (2016). KIF5B and Nup358 cooperatively mediate the nuclear import of HIV-1 during infection. PLoS Pathog.

[110] Procter DJ, Banerjee A, Nukui M, Kruse K, Gaponenko V, Murphy EA (2018). The HCMV assembly compartment is a dynamic Golgi-derived MTOC that controls nuclear rotation and virus spread. Dev Cell.

